# Effect of a probiotic formula on gastrointestinal health, immune responses and metabolic health in adults with functional constipation or functional diarrhea

**DOI:** 10.3389/fnut.2023.1196625

**Published:** 2023-07-10

**Authors:** Yanyi Zheng, Leiming Xu, Silu Zhang, Yanwen Liu, Jiayi Ni, Guoxun Xiao

**Affiliations:** ^1^Shenzhen Precision Health Food Technology Co., Ltd., Shenzhen, China; ^2^Department of Gastroenterology, Shanghai Jiaotong University Affiliated Xinhua Hospital, Shanghai, China; ^3^School of Bioengineering, East China University of Science and Technology, Shanghai, China; ^4^Sprim (China) Consulting Co. Ltd., Shanghai, China

**Keywords:** functional constipation, functional diarrhea, gastrointestinal health, immune responses, metabolic health, probiotics, Rome IV

## Abstract

**Objective:**

Our aim was to determine the efficacy of four-week probiotic supplementation on gastrointestinal health. The secondary objectives were to assess probiotic effects on immune reaction, as well as weight control and metabolic health.

**Methods:**

We conducted two randomized sub-trials, respectively, among subjects who were diagnosed with functional constipation (FC) or functional diarrhea (FDr) according to the Rome IV criteria. In each sub-trial, 70 eligible Chinese adults were randomized to receive a multi-strain probiotic combination or a placebo. Gastrointestinal symptoms, defecation habits, stool characteristics, blood and fecal biochemistry markers, anthropometrics measures, stress-associated responses, and intestinal flora changes were assessed at baseline and after probiotics intervention.

**Results:**

Four weeks of probiotic supplementation reduced overall gastrointestinal symptoms scores in FC participants (*p* < 0.0001). Their mean weekly stool frequency increased from 3.3 times to 6.2 times; immune response and inflammation markers improved with increases in serum IgA, IFN-γ and fecal sIgA, and decrease in hsCRP; most components of lipid profile were significantly ameliorated, with increases in HDL-C and reductions in TC and TG; body weight, body mass index and basal metabolic rate decreased following probiotics consumption. For FDr participants, probiotics consumption markedly reduced overall gastrointestinal symptom scores (*p* < 0.0001); decreased stool frequency by 3 times per week; increased IgA, IFN-γ, sIgA concentrations, while lowered hsCRP and IL-4 levels. Both FC and FDr participants had improvement in the scores of defecation habits, anxiety or depression, and perceived stress. Probiotics supplementation promoted the production of all three major short-chain fatty acids. No changes were observed in LDL-C, IgG, IgM, IL-8, IL-10 and motilin.

**Conclusion:**

Supplementation with the probiotic formula over a four-week period could help relieving gastrointestinal symptoms, improving satisfaction with defecation habits, emotional state and immune response, and ameliorating dysbacteriosis in participants with FC or FDr. It also had beneficial effects on lipid metabolism and weight control for FC participants.

## 1. Introduction

Functional bowel disorders (FBDs) are functional gastrointestinal (GI) disorders with symptoms attributable to the middle or lower GI tract (1), and involve chronic symptoms of bloating, abdominal pain, constipation or diarrhea. The FBDs have no identifiable morphologic and physiological abnormalities that can account for their defining symptoms (2). Diagnosis therefore relies on the patient’s interpretation and reporting of an illness experience, and it is classified primarily in terms of symptoms. The latest version of the most widely accepted diagnostic criteria, Rome IV, recognizes six different FBDs (3). Two common ones among them are functional constipation (FC) and functional diarrhea (FDr).

According to the Rome diagnosis criteria, FC presents as persistently difficult, infrequent, or seemingly incomplete defecation, and FDr is characterized by recurrent and urgent passage of loose or watery stools. Both FC and FDr exclude those that meet irritable bowel syndrome (IBS) criteria, although abdominal pain and/or bloating may be present (3). Deviation from normal intestinal transit frequency and stool characteristics are among the major symptoms of FC and FDr. A stool diary incorporating the Bristol Stool Form Scale is a reliable tool to verify stool form (4). Patients with FC may present with fewer than three self-bowel movement per week and/or hard stools (Bristol stool form scale 1–2) in more than a quarter of defecations. On contrary, occurrence of rapid bowel movement among patients with FDr increases the percentage of water in stool, reduces its viscosity and makes it hard to retain (5).

Global prevalence of FC ranged from 15.3% using the Rome I criteria to 10.1% when the Rome IV criteria were applied (6). A meta-analysis of studies from China reported 8.1% (95% confidence interval, CI: 5.6%–11.8%) of FC prevalence using the Rome IV criteria (7). FC affects all ages and is most common in women and non-whites (8). A cross sectional study in Iran has showed the association of FC with overweight; about 60% of FC patients had body mass index (BMI) greater than 25 kg/m^2^ (9). When FC becomes chronic, individuals risk developing mental and social comorbidities, including anxiety, depression and reductions in social and work activities diminish the person’s quality of life (10–12).

Reported prevalence rates for FDr in different countries range from 1.5% to 17% (13–17). In a recent multinational online survey in 54,127 individuals from 26 countries, the prevalence of Rome IV confirmed FDr was 4.7% (4.5%–4.9%), while it was 1.2% (1.0%–1.3%) in a household survey sample of 18,949 individuals from 9 countries (8). Chronic diarrhea caused by FDr is associated with various lower and upper GI symptoms, such as reflux, dyspepsia, heartburn, nausea, abdominal pain and bloating (18, 19). Risk factors of FDr include food sensitivity, irregular lifestyle and stress (20, 21).

The pathophysiology of FC and FDr is still not fully elucidated. A number of diverse mechanisms seem to contribute to symptom generation, including altered gut microbial environment, abnormal GI motility, brain-gut disturbances, genetic and environment factors, and psychosocial factors (22–24). Besides, a common link between FC and FDr may be related to inflammation in the gut (25–27). Increasing studies have indicated that the gut microbiota plays a key role in various activities of host physiology, including gut motility (28). Gut dysbiosis may contribute to the pathogenesis of FC and FDr due to the existence of the brain-gut-microbiome axis (29, 30). This potential association between gut microbiota perturbations and FC/FDr opens therapeutic possibilities, that is, to restore the gut microbiota balance by dietary microbial interventions. Probiotics are one of the most commonly used supplemental modalities and have shown beneficial effects in improving colonic transit and defecation frequency (31, 32) among patients with FC, as well as the potential to alleviate the symptoms of chronic diarrhea (33, 34). Nevertheless, few clinical studies have explored the effects of probiotics in both FC and FDr population. In addition, since the efficacy of probiotics is both strain-specific and outcome-specific (35), supplements with multiple strains may have additive or synergistic effects. The present study therefore aimed to investigate the effect of a multi-strain probiotics formula on the GI symptoms, gut inflammation, and psychological and physiological responses related to FC and FDr.

## 2. Materials and methods

### 2.1. Study design and participants

The study contains two parallel randomized trials in subjects with FC and those with FDr, respectively. Seventy subjects were enrolled in each sub-trial to ensure at least 60 complete the study, accounting for a 15% potential attrition rate. Subjects were randomized to two groups and were administered the study probiotic formula or a placebo over the period of 4 weeks (the intervention period). Subjects were followed for another week after the end of the intervention (the washout period) to assess the persist of the probiotic effect. The research practices of this study were in accordance with the Declaration of Helsinki. The Institutional Review Board of Shanghai Nutrition Society reviewed and approved this study. All subjects provided written informed consent prior to enrollment.

Eligible subjects included males and females aged 18–65 years, self-reported history of constipation or diarrhea symptoms, and fulfilled the Rome IV criteria for FC or FDr (36). To investigate the weight control effect of probiotics in subjects with FC, we included those with body mass index (BMI) over 24 kg/m^2^. We excluded subjects who were under treatment for GI symptoms; lactose intolerant; currently suffering from organic diseases that might affect intestinal function, such as prior GI resection, colon or rectal cancer, inflammatory bowel disease, diabetes, hyperthyroidism or hypothyroidism, Hirschsprung disease, scleroderma, anorexia nervosa; on diet, doing excessive workout, taking weight control drugs or drugs that can affect their appetite in the past 3 months; having history or clinically diagnosed with any diseases that may affect efficacy evaluation of the study product, including GI disorders, liver, kidney, endocrine, blood, respiratory and cardiovascular diseases; currently or have been overusing alcohol, drugs or supplements which may cause intestinal dysfunction or affect efficacy evaluation of the study product; frequently using drugs that may affect GI function or the immune system; used laxatives or other supplements to improve digestive function within 2 weeks before study entry; used dairy products or other products that contains prebiotics or probiotics within 10 days before study entry; pregnant or breast-feeding women. Subjects were required to avoid the consumption of other fermented milk, yogurt and supplements containing probiotic or prebiotic, and to maintain their usual diet and daily physical exercise habits.

### 2.2. Study product

The active probiotic combination under study was supplied by WONDERLAB® (Shenzhen, China) as a fine white powder (2 g) packed in sealed bottle. The product contained 4 × 10^10^ colony forming units of six probiotic strains: *Lactobacillus (L) acidophilus* NCFM, *Bifidobacterium (B) lactis* HN019, *B. lactis* Bl-04, *B. lactis* B420, *Lactobacillus (L) plantarum* Lp-115 and *Lacticaseibacillus (L) paracasei* Lpc-37, with addition of prebiotics. The placebo contained only maltodextrin and the appearance was the same as the probiotic product. Each participant orally received the products directly or with warm water half an hour after meal, twice daily, for a period of 4 weeks.

### 2.3. Efficacy evaluation

The primary outcome was a GI symptom score assessed via the Intestinal Health Evaluation Form (37). At the end of each week, subjects assessed the frequency/severity of their GI symptoms, including bloating, abdominal pain, early feeling of fullness, belching, poor appetite, heavy stomach, dyspepsia, nausea or vomiting, poor GI motility, and dissatisfaction about digestive function. Each symptom was rated on a 4-point Likert-type scale, as asymptomatic (score = 0), seldom/mild (score = 1), often/moderate (score = 2), or always/severe, affecting daily life (score = 3). The overall GI symptom score was obtained by summing up all the individual scores.

Evaluations of defecation habits in the past week were recorded on the Defecation Evaluation Form (37). It gathered information in two areas: (a) scoring for intestinal health, including four individual item scores for frequency of defecation, difficulty in defecation, time of defecation and simultaneous phenomenon of defecation; and (b) scoring for satisfaction with defecation habit, including four individual item scores for the satisfaction with frequency of defecation, time of defecation, stool characteristics and overall defecation habit. Each individual item score ranges from 0 to 3, with lower score indicated better intestinal health or satisfaction with defecation habit. The overall scores for intestinal health and satisfaction with defecation habit were the sum of the individual scores in each area.

Weekly stool frequency was recorded. Stool characteristics were evaluated using the Bristol Stool Form Scale (4). The Bristol scale of stool consistency is a visual medical aid designed to classify the form of human feces into seven groups: type 1 stool, separate hard lumps, like nuts, hard to pass; Type 2, sausage-shaped, but lumpy; Type 3, like a sausage but with cracks on its surface; Type 4, like a sausage or snake, smooth and soft; Type 5, soft blobs with clear cut edges (passed easily); Type 6, fluffy pieces with ragged edges, a mushy stool; and Type 7, watery, with or without solid pieces. For stool color assessment, seven colors close to the actual color of the stools were printed on the questionnaire and subjects were requested to select one of them closest to their stool color. The score for stool color ranges from 1 to 7, with larger score indicating darker stool color.

Body weight, height, body mass index (BMI), waist and hip circumference, body fat percentage and basal metabolic rate (BMR) were measured using InBody bioelectrical body composition analyzing device (InBody Co., Ltd., Seoul, South Korea). Blood samples were drawn between 8 to 10 a.m. following an overnight fast of at least 12 h to quantify biomarkers of lipid concentrations, systemic inflammation, immune response and GI motility. The biomarkers measured include total cholesterol (TC), triglycerides (TG), high-density lipoprotein cholesterol (HDL-C), low-density lipoprotein cholesterol (LDL-C), high-sensitivity C-reactive protein (hsCRP), immunoglobulin A (IgA), immunoglobulin G (IgG), and immunoglobulin M (IgM), interleukin-4, -8 and -10 (IL-4, IL-8, and IL-10), interferon-γ (IFN-γ) and motilin (MTL). Fecal secretory immunoglobulin A (sIgA) and short-chain fatty acids (SCFAs) and were also assessed as these are sensitive markers for inflammation in the GI tract.

Participants completed the Hospital Anxiety and Depression (HAD) questionnaire and the Perceived Stress Scale (PSS) questionnaire to investigate the effects of the study formula on anxiety, depression, and perceived stress. The Chinese language versions were used in all cases. The HAD is a self-assessment scale for measuring states of anxiety and depression (38). The questionnaire comprises seven questions for anxiety and seven questions for depression, with points of each question ranging from 0 to 3. The overall HAD score was obtained by summing the scores of 14 questions. Higher score indicates more severe anxiety or depression. The PSS consists 14 items intended to measure the degree to which individuals perceived their life circumstances as stressful within the last month (39). Individuals rate items on a 5-point Likert scale, ranging from 0—“Never” to 4—“Very often.” The total score was calculated by summing the scores of the 14 items.

The scores for GI symptoms, intestinal health and satisfaction with defecation habits were measured at baseline and at the end of each week during the study (week 1 to week 5). The InBody test, blood and fecal laboratory tests, HAD and PSS scales were assessed at baseline and the end of the intervention (week 4).

Participants were instructed to record their daily food and beverage intake during the 3 days before the baseline, the last 3 days before the end of the intervention and the last 3 days of the washout period according to the food models and scales provided. The portion sizes were converted to grams or milliliters and summarized by food categories. Physical activity in the past week of the baseline, in the last week of the intervention period and in the washout week were assessed using a continuous measure for metabolic equivalent of task (MET-minutes) derived using the algorithms provided by the Guidelines for Data Processing and Analysis of the International Physical Activity Questionnaire (IPAQ) (40). These data were collected to assess if there was a change in diet and exercise frequency.

Gut microbial diversity was monitored using 16 s rRNA sequencing (41) of the stool samples collected from a random subsample of 30 subjects, stratified by intervention groups, in each sub-trail at baseline, the end of week 4 and week 5. The within-group changes from baseline in amplicon sequence variants (ASV) relative abundance of Bifidobacterium and Lactobacillus was used to evaluate the survival of the supplied probiotics during GI passage. The abundance was analyzed on log10 scale and the results were back-transformed. This analysis was performed for the pooled subsample of FC and FDr subjects. For exploratory purpose, we summarized the relative abundance of the 20 most abundant ASVs to assess change of gut microbiota composition, especially in genus Klebsiella, Prevotella, Escherichia-Shigella, Bacteroides and Blautia following probiotics supplementation.

### 2.4. Statistical analysis

Demographic and baseline characteristics were summarized by study group. Continuous outcome variables are reported as means ± SD or median (quartiles), and categorical outcomes are reported as frequency (%). Prior to testing, distributional assumptions for the outcomes were assessed and transformations or nonparametric versions of the tests were used if deemed necessary. The differences between study groups were evaluated using analysis of (co)variance for normal distributed outcomes. Analyses of post-intervention data were adjusted for baseline measurements. For non-normal data, group differences were analyzed using Kruskal Wallis test. The differences between baseline and post-intervention outcomes within each study group were evaluated using paired t-test for normal distributed continuous outcomes and Wilcoxon signed ranks test for non-normal or ordinal outcomes. A mixed model was used to assess repeated measured outcomes. The number and percent of adverse events (AEs) and serious adverse events (SAEs) were summarized. All analyses were conducted for enrolled participants who consumed at least one dose of the study product. The significance level for the statistical tests was set at 0.05. Statistical analyses were performed using SAS software version 9.4 (SAS Institute Inc., Cary, NC, United States).

## 3. Results

A total of 140 subjects participated in this study, with 70 in the each of the two sub-trails. Of all enrolled subjects, mean age was 45.7 years (standard deviation, SD: 11.5 years) and 28.6% were men (Table 1). All subjects have initiated product consumption and were included in the analyses. In the FC trail, 7 (10.0%) subjects withdrew early (2 in the probiotic group and 5 in the control group [CG]); while in the FDr trail, 7 (10%) subjects withdrew early (3 in the probiotic group and 4 in the CG). All early withdraws were due to personal reasons and were not related to the study products. Baseline characteristics were comparable between the study arms in each sub-trail. During the study period, all participants complied with dietary restrictions and maintained similar diet (Supplementary Table 1) and physical activity levels (Supplementary Table 2).

**Table 1:** Baseline characteristics.

		Functional constipation			Functional diarrhea		
		Probiotic (*n* = 35)	Control (*n* = 35)	Group difference *p*-value	Probiotic (*n* = 35)	Control (*n* = 35)	Group difference *p*-value
Gender	Male	10 (28.6%)	10 (28.6%)	1.000	10 (28.6%)	10 (28.6%)	1.000
	Female	25 (71.4%)	25 (71.4%)		25 (71.4%)	25 (71.4%)	
Age, year		46.4 ± 12.8	47.6 ± 10.7	0.686	43.1 ± 11.8	45.7 ± 10.6	0.331
Body weight, kg		73.2 ± 11.4	71.9 ± 12.1	0.666	61.9 ± 11.0	63.5 ± 11.1	0.538
Height, cm		165.0 ± 7.3	163.7 ± 8.9	0.498	164.7 ± 9.0	163.9 ± 8.0	0.692
Body mass index (BMI), kg/m^2^		26.7 ± 2.7	26.7 ± 2.5	0.926	22.7 ± 3.0	23.6 ± 3.1	0.253
Systolic blood pressure, mmHg		127.2 ± 14.8	130.3 ± 17.4	0.431	118.5 ± 15.7	122.2 ± 15.8	0.335
Diastolic blood pressure, mmHg		79.8 ± 12.4	79.4 ± 10.8	0.886	76.1 ± 14.9	75.4 ± 11.6	0.817

Data are presents as mean ± standard deviation or frequency (%). Group differences were evaluated using one-way analysis of variance for continuous variables and chi-square test for categorical variables.

### 3.1. Gastrointestinal symptoms

The GI symptoms scores of both the FC (−4.0 points; 95% confidence interval, CI: −5.2, −2.7 points) and the FDr (−3.5 points; −4.7, −2.4) subjects significantly decreased since the second week of probiotics consumption and were both significantly lower than that of the CGs (*p* = 0.0001 and 0.0003, respectively, Table 2). The scores in both groups further reduced as the intervention continued until the end of week 4 (FC: −4.9 points; −6.2, −3.7 and FDr: −4.2 points; −5.4, −3.0), which were significantly lower than the CGs (*p* < 0.0001). After the intervention ended for a week, the symptoms scores of the probiotic groups remained significantly lower compared to that of the baseline and that of the CGs.

**Table 2:** Gastrointestinal symptoms scores.

Visit	Functional constipation			Functional diarrhea		
	Probiotic	Control	Group difference *p*-value	Probiotic	Control	Group difference *p*-value
Baseline	11.9 ± 4.6	11.6 ± 4.3	0.785	9.5 ± 4.6	9.4 ± 4.3	0.952
Week 1	10.7 ± 4.7	11.8 ± 3.7	0.288	8.5 ± 4.0	9.2 ± 3.4	0.431
Week 2	7.9 ± 5.5	11.9 ± 3.4	0.0001	6.0 ± 4.6	9.5 ± 3.5	0.0003
Week 3	7.3 ± 4.9	12.2 ± 2.7	<0.0001	6.0 ± 4.2	9.4 ± 3.1	0.0004
Week 4	7.0 ± 5.7	12.0 ± 3.1	<0.0001	5.3 ± 4.5	9.6 ± 3.1	<0.0001
Week 5	7.2 ± 5.0	12.4 ± 3.3	<0.0001	5.7 ± 4.3	9.7 ± 3.4	<0.0001

	Difference	*p*-value	Difference	*p*-value	Difference	*p*-value	Difference	*p*-value
Week 1 vs. baseline	−1.2 (−2.5, 0.03)	0.056	0.2 (−1.1, 1.4)	0.789	−1.1 (−2.2, 0.1)	0.076	−0.3 (−1.4, 0.9)	0.665
Week 2 vs. baseline	−4.0 (−5.2, −2.7)	<0.0001	0.4 (−0.9, 1.7)	0.541	−3.5 (−4.7, −2.4)	<0.0001	0.04 (−1.2, 1.3)	0.944
Week 3 vs. baseline	−4.6 (−5.9, −3.3)	<0.0001	0.7 (−0.7, 2.0)	0.319	−3.5 (−4.7, −2.4)	<0.0001	−0.1 (−1.3, 1.2)	0.934
Week 4 vs. baseline	−4.9 (−6.2, −3.7)	<0.0001	0.5 (−0.8, 1.8)	0.450	−4.2 (−5.4, −3.0)	<0.0001	0.2 (−1.1, 1.4)	0.814
Week 5 vs. baseline	−4.7 (−6.0, −3.4)	<0.0001	0.9 (−0.4, 2.2)	0.179	−3.9 (−5.0, −2.7)	<0.0001	0.2 (−1.0, 1.4)	0.774
Week 5 vs. week 4	0.3 (−1.0, 1.5)	0.689	0.4 (−1.0, −1.8)	0.564	0.3 (−0.8, 1.5)	0.564	0.03 (−1.2, 1.3)	0.959

Data presented are mean ± standard deviation. Between and within-group differences were evaluated using repeated measured analysis of variance.

**Figure 1:**
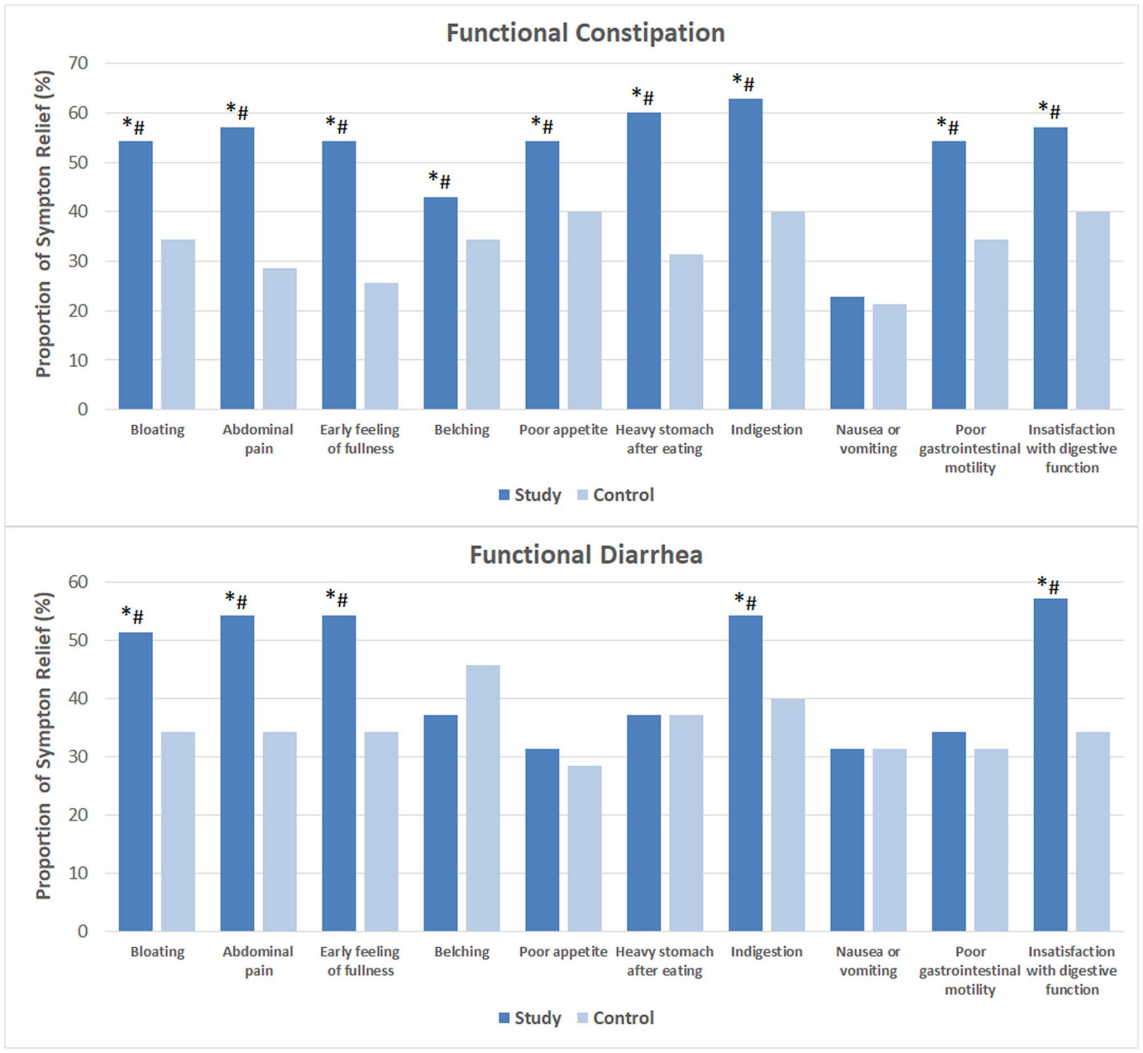
Relief of gastrointestinal symptoms after 4 weeks of intervention. The mean values were plotted. Between and within-group differences were evaluated using repeated measures logistic regression. ^*^Significant difference compared to control, *p*-values range from 0.0003 to 0.049; ^#^Significant difference compared to baseline, *p*-values range from <0.0001 to 0.036.

Figure 1 shows the proportion of subjects that noticed relief of each individual GI symptom. Following 4 weeks of probiotics consumption, heavy stomach after eating and dyspepsia were markedly reduced in over 60% of the FC subjects. More than half of these subjects had relived symptoms of bloating, abdominal pain, early feeling of fullness, poor appetite and poor GI motility, as well as improved satisfaction with their digestive function. In addition, 42.9% reported relief of belching. While 22.9% reported less severe nausea or vomiting, the difference compared to baseline and the CG was not significant. Among the majority of FDr subjects, probiotics consumption effectively relieved bloating, abdominal pain, early feeling of fullness, dyspepsia and improved satisfaction with digestive function.

### 3.2. Intestinal health

**Figure 2:**
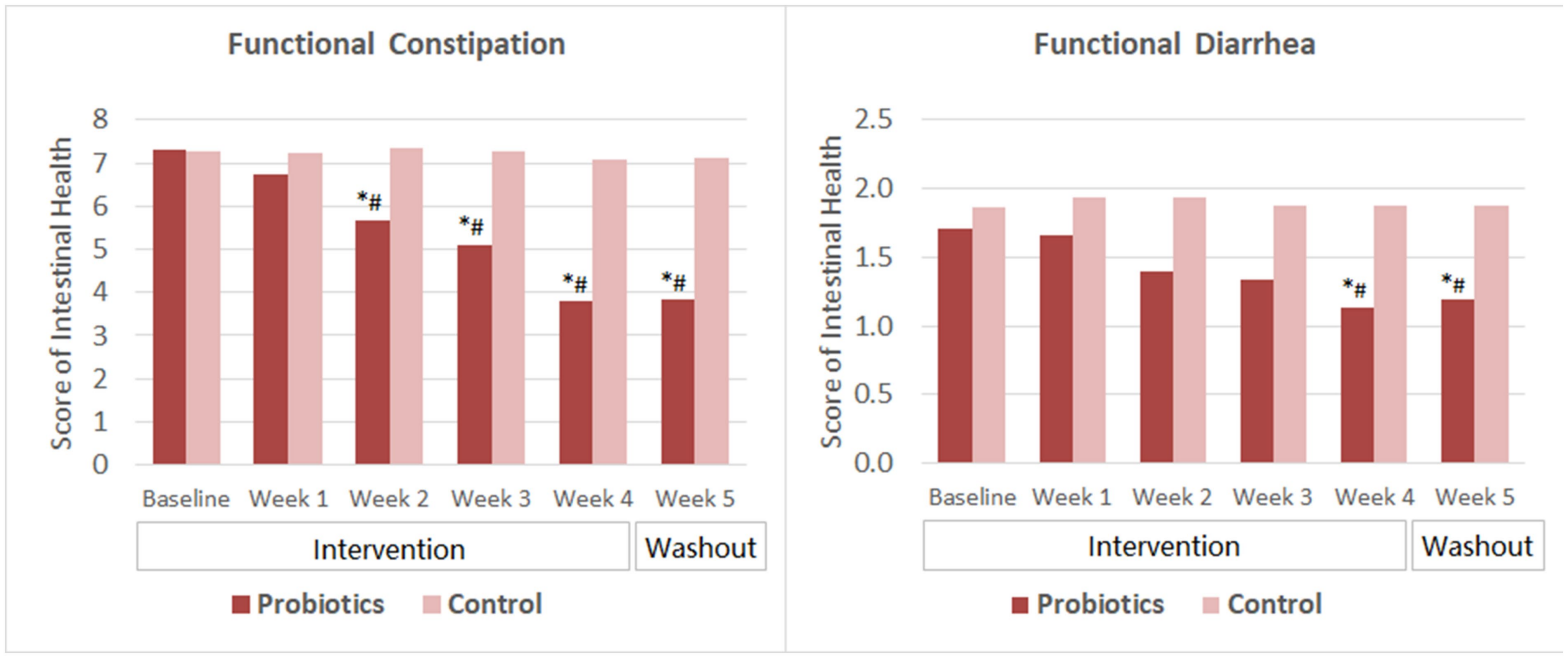
Scores of intestinal health. The mean values were plotted. Between and within-group differences were evaluated using repeated measures analysis of variance. ^*^Significant difference compared to control, *p*-values range from <0.0001 to 0.025; ^#^Significant difference compared to baseline, *p*-values range from <0.0001 to 0.002.

Subjects with FC reported significant improvement in intestinal health (indicated by lower score) since the second week of probiotics consumption. The score further reduced as the intervention continued and was significantly lower than that of the CG since week 2. The mean score remained at the same level as the intervention ended for 1 week (Figure 2).

For subjects with FDr, the overall score reduced significantly from baseline and was significantly lower than that of the CG after 4 weeks of probiotics consumption. At the end of the washout period, the mean score slightly increased but was still significantly lower than that of the baseline and the CG. The change of scores from week 4 to week 5 was not significant in the probiotics group.

### 3.3. Satisfaction of defecation habit

**Figure 3:**
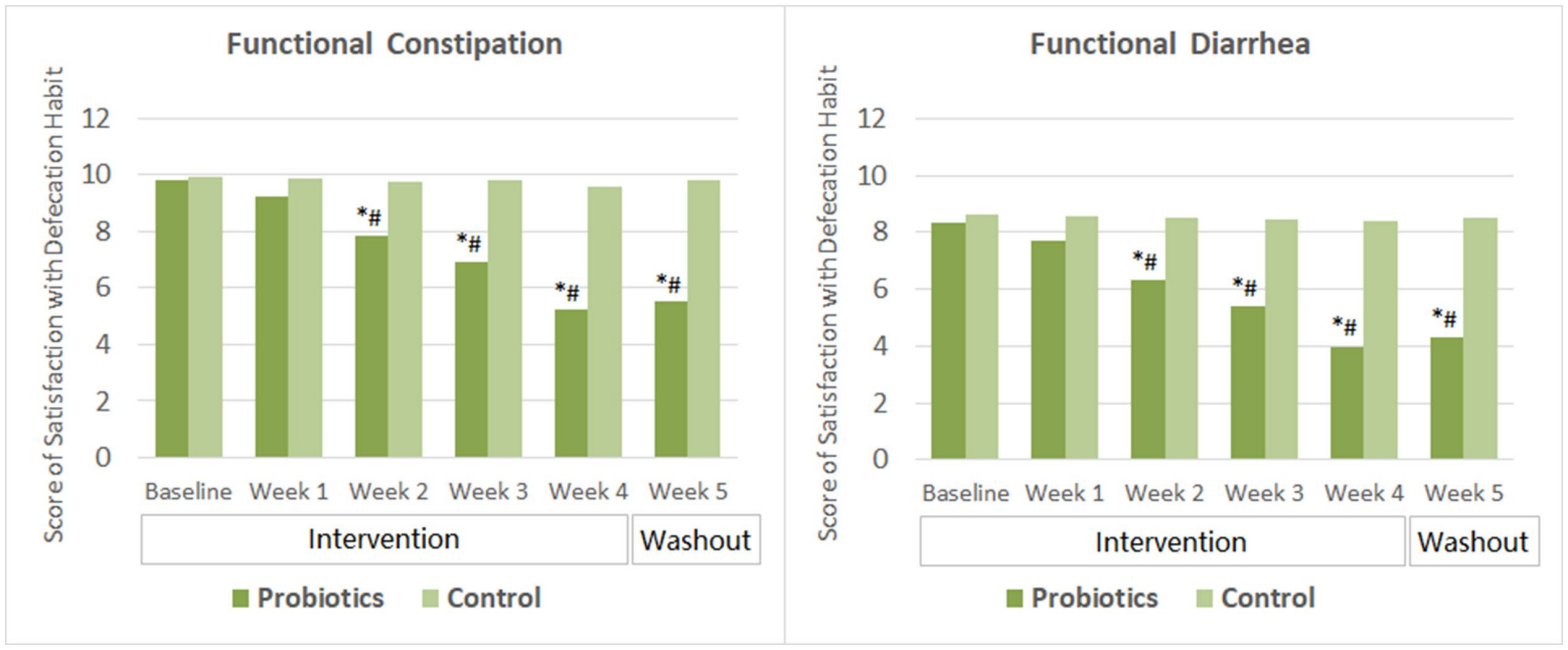
Scores of satisfaction with defecation habit. The mean values were plotted. Between and within-group differences were evaluated using repeated measures analysis of variance. ^*^Significant difference compared to control, *p*-values range from <0.0001 to 0.027; ^#^Significant difference compared to baseline, *p*-values range from <0.0001 to 0.016.

After 2 weeks of probiotics supplementation, significant improvement in the satisfaction with defecation habit (indicated by decreased score) was observed among subjects with FC and those with FDr as well. Subjects’ satisfaction score reduced further as taking the probiotics product for two more weeks. By the end of the washout period, the satisfaction scores of the probiotics group in both the FC and the FDr sub-trials were significantly lower than their own baseline scores, and also showed no significant change compared to that at the end of the intervention. The differences in the satisfactions scores between the probiotics group and the CG were significant from week 2 to week 5 in both sub-trials (Figure 3).

### 3.4. Stool frequency and Bristol scores of stool characteristics

**Figure 4:**
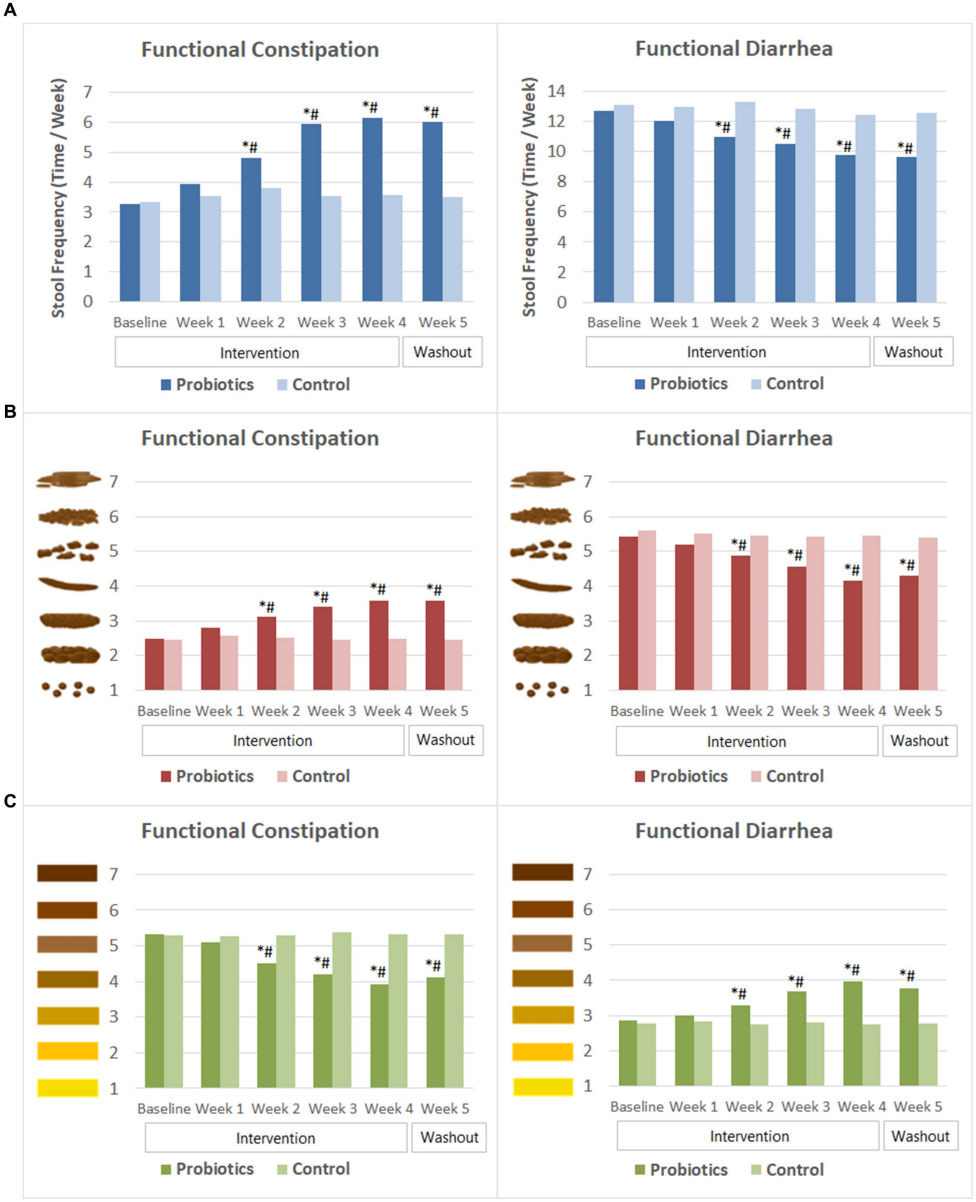
Weekly stool frequency and Bristol scores of stool characteristics. **(A)** Stool frequency. **(B)** Bristol scores of stool consistency. **(C)** Bristol scores of stool color. The mean values were plotted. Between and within-group differences were evaluated using repeated measures analysis of variance. ^*^Significant difference compared to control, *p*-values range from <0.0001 to 0.037; ^#^Significant difference compared to baseline, *p*-values range from <0.0001 to 0.015.

Subjects with FC had an average stool frequency of 3.3 ± SD 1.4 times per week at baseline. Their stool frequency increased significantly after 2 weeks of probiotics consumption and continued to improve in the following 2 weeks. By the end of probiotics intervention, the average stool frequency reached 6.2 ± 1.5 times per week. At the end of the washout period, their stool frequency maintained at about 6 times per week. In the meantime, significant improvement was observed in Bristol scores of stool characteristics. The average stool consistency of the probiotics group changed from lumpy and sausage like (type 2, mild constipation) at baseline to normal consistency of type 3 or type 4 since the second week of probiotics consumption. Moreover, their stool color become significantly lighter compared to that at baseline since week 2. The probiotics group had significantly higher weekly stool frequency, less dry and lighter colored stool than that of the CG from week 2 to week 5 of the study (Figure 4).

In the FDr sub-trial, subjects experienced multiple times of defecation in 1 day at baseline. Their weekly stool frequency decreased gradually following probiotics consumption. At week 2, stool frequency of the probiotics group was 11.0 ± 4.1 times per week, which was significantly lower than that at baseline (12.7 ± 4.0 times) and that of the CG (13.3 ± 3.7 times). The mean stool frequency reduced to 9.8 ± 4.7 times per week by the end of the probiotics intervention and remained similar after 1 week of washout. Stool consistency and stool color also improved after probiotics consumption. Bristol score of stool consistency reduced significantly from 5.4 ± 0.5 at baseline to 4.9 ± 1.1 at week 2, and then to 4.2 ± 0.8 at the end of the intervention. The score of stool color increased significantly from baseline since the second week of probiotics consumption, indicating that the stool color became darker. The differences in stool frequency, Bristol scores of stool consistency and stool color between the probiotics group and the CG were significant from week 2 to week 5 (Figure 4).

### 3.5. HAD and PSS scores

For the probiotics group with FC, both HAD scores (*p* = 0.001) and PSS score (*p* = 0.013) significantly decreased from baseline to end of the intervention and were significantly lower than that of the CG, indicating a significant effect of the probiotic formula in reducing anxiety, depression and perceived stress. After 1 week of washout, both scores of the probiotics group remained at similar level and were significantly lower than the baseline scores and that of the CG (Table 3).

**Table 3:** HAD and PSS scores.

Visit	Functional constipation					Functional diarrhea				
	Probiotics		Control		Group difference *p*-value	Probiotics		Control	Group difference *p*-value	
**HAD score**										
Baseline	12.1 ± 5.9		12.3 ± 5.3		0.847	12.9 ± 5.4		12.8 ± 5.5		0.913
Week 4	8.5 ± 4.7		12.3 ± 4.6		0.002	9.0 ± 4.6		12.9 ± 4.6		0.001
	**Difference**	**p-value**	**Difference**	**p-value**		**Difference**	**p-value**	**Difference**	**p-value**	
Week 4 vs. baseline	−3.2 (−4.9, −1.5)	0.001	0.5 (−0.5, 1.6)	0.301		−4.3 (−6.3, −2.2)	0.0002	−0.1 (−1.0, 0.8)	0.885	
**PSS score**										
Baseline	36.6 ± 9.4		36.6 ± 7.8		0.978	36.3 ± 8.7		36.4 ± 7.2		0.976
Week 4	32.6 ± 8.3		36.1 ± 5.1		0.047	33.3 ± 6.5		37.4 ± 6.7		0.015
	**Difference**	**p-value**	**Difference**	**p-value**		**Difference**	**p-value**	**Difference**	**p-value**	
Week 4 vs. baseline	−3.9 (−6.9, −0.9)	0.013	0.7 (−0.8, 2.2)	0.357		−3.7 (−5.9, −1.4)	0.002	1.1 (−0.8, 3.0)	0.258	

HAD, Hospital Anxiety and Depression Scale; PSS, Perceived Stress Scale. Data are presented as mean ± standard deviation. Differences between study groups at each visit were evaluated using analysis of (co)variance. Analysis of post-intervention data adjusted for baseline measurements. Within-group differences between post-intervention and baseline are presented as the difference of least-squares means (95% confidence interval) and evaluated using paired *t*-test.

Probiotics supplementation showed similar effect in relieving anxiety, depression and perceived stress among subjects with FDr. The probiotics group reported a significant lower HAD score (*p* = 0.0002) and PSS score (*p* = 0.002) at the end of the intervention compared to that at baseline and the CG (Table 3).

### 3.6. Blood and fecal biomarkers

Among FC subjects, there were significant reductions in serum level of TC (*p* = 0.003), TG (*p* < 0.0001) and hsCRP (*p* < 0.0001) after 4 weeks of probiotics consumption. Besides, probiotic consumption resulted in significant increases in serum HDL-C (*p* = 0.005), IgA (*p* = 0.0004), IFN-γ (*p* = 0.0003), as well as fecal concentrations of sIgA (*p* < 0.0001), acetic acid (*p* < 0.0001), propanoic acid (*p* < 0.0001) and butyric acid (*p* < 0.0001). The difference between the probiotics group and the CG was significant in the above-mentioned biomarkers. There was no significant change in LDL-C, IgG, IgM, IL-4, IL-8, IL-10, and motilin levels (Table 4).

**Table 4:** Blood and fecal biomarkers.

Outcome	Functional constipation			Functional diarrhea		
	Probiotics	Control	Group difference *p*-value	Probiotics	Control	Group difference *p*-value
**Baseline**						
TC, mmol/L	5.2 ± 0.6	5.2 ± 0.6	0.842	Not measured		
TG, mmol/L	1.6 ± 0.7	1.7 ± 0.9	0.907			
HDL-C, mmol/L	1.5 ± 0.3	1.5 ± 0.2	0.617			
LDL-C, mmol/L	2.9 ± 0.6	2.8 ± 0.7	0.859			
hsCRP, mg/L	4.6 ± 1.5	4.5 ± 1.4	0.743	4.1 ± 1.9	4.1 ± 1.4	0.947
IgA, g/L	2.2 ± 0.7	2.2 ± 0.6	0.897	1.9 ± 0.6	1.9 ± 0.6	0.834
IgG, g/L	12.2 ± 2.6	12.3 ± 2.6	0.969	12.4 ± 2.8	12.4 ± 2.1	0.918
IgM, g/L	1.1 ± 0.5	1.1 ± 0.5	0.817	1.1 ± 0.5	1.1 ± 0.5	0.888
IL-4, pg./mL^a^	1.2 (0.79, 1.5)	1.3 (0.7, 1.7)	0.972	1.6 (0.9, 1.8)	1.5 (0.8, 1.8)	0.581
IL-8, pg./mL^a^	20.6 (15.2, 35.5)	22.0 (15.8, 39.1)	0.842	28.3 (16.3, 44.5)	28.7 (14.8, 39.9)	0.972
IL-10, pg./mL	23.6 ± 3.1	23.5 ± 2.8	0.851	23.9 ± 3.2	23.7 ± 3.0	0.790
IFN-γ, ng/L	18.8 ± 6.5	19.2 ± 7.7	0.832	16.5 ± 6.5	16.9 ± 6.3	0.797
Motilin, pg./mL^a^	39.4 (26.4, 69.6)	45.9 (32.2, 64.9)	0.577	68.5 (53.2, 87.2)	71.2 (58.2, 86.3)	0.865
sIgA, mg/dL	431.2 ± 260.5	423.5 ± 240.7	0.897	357.7 ± 280.4	365.0 ± 286.5	0.914
Acetic acid, mg/g	3.8 ± 0.5	3.8 ± 0.5	0.885	3.9 ± 0.4	3.9 ± 0.5	0.857
Propanoic acid, mg/g	1.4 ± 0.1	1.4 ± 0.2	0.892	1.4 ± 0.2	1.4 ± 0.2	0.482
Butyric acid, mg/g	0.7 ± 0.1	0.7 ± 0.1	1.000	0.7 ± 0.2	0.7 ± 0.1	0.841
**Week 4**						
TC, mmol/L	4.9 ± 0.6	5.1 ± 0.4	0.038	Not measured		
TG, mmol/L	1.4 ± 0.4	1.7 ± 0.8	0.038			
HDL-C, mmol/L	1.6 ± 0.3	1.5 ± 0.2	0.045			
LDL-C, mmol/L	2.7 ± 0.6	2.9 ± 0.5	0.411			
hsCRP, mg/L	2.8 ± 1.3	4.6 ± 1.3	<0.0001	3.1 ± 1.2	4.1 ± 1.5	0.005
IgA, g/L	2.6 ± 0.9	2.1 ± 0.7	0.041	2.1 ± 0.6	1.8 ± 0.6	0.043
IgG, g/L	12.4 ± 2.2	12.3 ± 2.7	0.846	12.8 ± 2.7	12.4 ± 2.4	0.526
IgM, g/L	1.2 ± 0.6	1.1 ± 0.5	0.730	1.1 ± 0.5	1.1 ± 0.5	0.877
IL-4, pg./mL^a^	1.1 (0.9, 1.4)	1.1 (0.9, 1.7)	0.429	1.2 (0.7, 1.4)	1.4 (0.9, 1.9)	0.048
IL-8, pg./mL^a^	18.9 (14.7, 33.5)	23.2 (13.9, 40.1)	0.810	27.6 (15.7, 40.0)	29.7 (19.8, 39.2)	0.967
IL-10, pg./mL	23.0 ± 2.9	23.5 ± 3.1	0.530	22.9 ± 3.0	23.5 ± 3.1	0.428
IFN-γ, ng/L	24.6 ± 6.4	19.6 ± 6.8	0.004	22.6 ± 6.2	16.9 ± 6.4	0.001
Motilin, pg./mL^a^	57.7 (46.2, 67.6)	55.0 (29.3, 77.4)	0.457	65.1 (55.8, 73.2)	73.3 (59.5, 83.9)	0.145
sIgA, mg/dL	569.5 ± 331.7	417.6 ± 248.09	0.045	510.0 ± 297.4	358.9 ± 297.0	0.048
Acetic acid, mg/g	4.2 ± 0.6	3.8 ± 0.51	0.009	4.3 ± 0.6	3.9 ± 0.4	0.002
Propanoic acid, mg/g	1.6 ± 0.2	1.4 ± 0.20	0.0002	1.6 ± 0.2	1.4 ± 0.3	0.002
Butyric acid, mg/g	0.8 ± 0.2	0.7 ± 0.10	0.006	0.8 ± 0.1	0.7 ± 0.2	0.036

Week 4 vs. baseline	Difference	*p*-value	Difference	*p*-value	Difference	*p*-value	Difference	*p*-value
TC, mmol/L	−0.3 (−0.5, −0.1)	0.003	0.1 (−0.1, 0.2)	0.233	Not measured			
TG, mmol/L	−0.3 (−0.5, −0.2)	<0.0001	0.1 (−0.01, 0.1)	0.103				
HDL-C, mmol/L	0.1 (0.04, 0.2)	0.005	0.01 (−0.1, 0.1)	0.851				
LDL-C, mmol/L	−0.1 (−0.3, 0.03)	0.117	0.1 (−0.1, 0.2)	0.220				
hsCRP, mg/L	−1.9 (−2.3, −1.4)	<0.0001	0.02 (−0.3, 0.3)	0.854	−1.1 (−1.7, −0.5)	0.001	−0.1 (−0.4, 0.2)	0.624
IgA, g/L	0.3 (0.2, 0.5)	0.0004	−0.04 (−0.1, 0.04)	0.353	0.2 (0.1, 0.4)	0.0003	−0.02 (−0.1, 0.1)	0.699
IgG, g/L	0.1 (−0.3, 0.4)	0.755	0.03 (−0.4, 0.4)	0.860	0.5 (−0.02, 1.0)	0.061	−0.04 (−0.6, 0.5)	0.867
IgM, g/L	0.1 (−0.1, 0.2)	0.509	−0.02 (−0.1, 0.1)	0.774	0.1 (−0.02, 0.1)	0.155	0.01 (−0.1, 0.1)	0.821
IL-4, pg./mL^a^	−0.02 (−0.3, 0.1)	0.248	0.1 (−0.1, 0.2)	0.182	−0.2 (−0.4, 0.1)	0.009	−0.1 (−0.2, 0.2)	0.454
IL-8, pg./mL^a^	−1.5 (−6.4, 6.7)	0.637	1.1 (−6.4, 5.6)	0.952	−2.4 (−6.6, 4.0)	0.281	−1.8 (−4.7, 7.7)	0.826
IL-10, pg./mL	−0.7 (−2.2, 0.8)	0.355	0.1 (−1.2, 1.4)	0.841	−1.1 (−2.5, 0.3)	0.118	−0.4 (−1.7, 1.0)	0.559
IFN-γ, ng/L	5.6 (2.8, 8.5)	0.0003	−0.1 (−1.9, 1.8)	0.957	6.2 (4.0, 8.5)	<0.0001	−0.1 (−1.0, 0.8)	0.833
Motilin, pg./mL^a^	12.0 (−12.1, 25.1)	0.206	−1.1 (−11.9, 14.6)	0.802	−1.0 (−20.7, 11.6)	0.475	2.0 (−13.8, 7.6)	0.660
sIgA, mg/dL	131.5 (77.1, 185.9)	<0.0001	−13.7 (−47.2, 19.8)	0.411	134.5 (69.9, 199.2)	0.0002	−4.8 (−35.1, 25.5)	0.748
Acetic acid, mg/g	0.4 (0.3, 0.5)	<0.0001	0.02 (−0.1, 0.1)	0.551	0.4 (0.2, 0.6)	<0.0001	0.04 (−0.1, 0.2)	0.501
Propanoic acid, mg/g	0.2 (0.1, 0.2)	<0.0001	−0.01 (−0.1, 0.1)	0.818	0.2 (0.1, 0.2)	<0.0001	−0.01 (−0.1, 0.1)	0.734
Butyric acid, mg/g	0.1 (0.04, 0.1)	<0.0001	−0.01 (−0.03, 0.02)	0.708	0.1 (0.04, 0.1)	<0.0001	0.01 (−0.03, 0.1)	0.681

TC, total cholesterol; TG, triglycerides; HDL-C, high-density lipoprotein cholesterol; LDL-C, low-density lipoprotein cholesterol; hsCRP, high-sensitivity C-reactive protein; Ig, immunoglobulin; IL, interleukin; IFN-γ, interferon-γ; sIgA, secretory immunoglobulin A; Q1, the first quartile; Q3, the third quartile. Unless otherwise stated, data are presented as mean ± standard deviation. Differences between study groups at each visit were evaluated using analysis of (co)variance. Analysis of post-intervention data adjusted for baseline measurements. Within-group differences between post-intervention and baseline measurements are presented as mean (95% CI) and evaluated by paired *t*-test.

a. Data are presented as median (Q1, Q3). Differences between study groups at each visit were evaluated using Kruskal Wallis test. Differences between post-intervention and baseline measurements are presented as median (Q1, Q3) and evaluated by Wilcoxon signed rank test.

Among FDr subjects, significant lower levels of hsCRP (*p* = 0.001) and IL-4 (*p* = 0.009), as well as significant higher levels of IgA (*p* = 0.0003), IFN-γ (*p* < 0.0001), fecal sIgA (*p* = 0.0002), acetic acid (*p* < 0.0001), propanoic acid (*p* < 0.0001) and butyric acid (*p* < 0.0001) were observed following 4 weeks of probiotics consumption. The probiotics group distinguished itself from the CG in the above-mentioned biomarkers post study intervention.

### 3.7. Anthropometric for subjects with functional constipation

Mean body weight decreased by 2.2 kg and BMI by 0.8 kg/m^2^ after four-week probiotics consumption, which were significantly lower than that at the baseline (both *p* = 0.001). The probiotics group also showed a 0.7 cm and 0.6 cm reduction in waist and hip circumference (*p* = 0.079 and 0.086, respectively) compared to the baseline (Table 5).

**Table 5:** Anthropometrics for subjects with functional constipation.

Outcome	Probiotics	Control	Group difference
			*p*-value
**Baseline**			
Height, cm	165.0 ± 7.3	163.7 ± 8.9	0.498
Body weight, kg	73.2 ± 11.4	71.9 ± 12.1	0.666
BMI, kg/m^2^	26.7 ± 2.7	26.7 ± 2.5	0.926
Waist circumference, cm	89.7 ± 9.5	89.1 ± 10.0	0.824
Hip circumference, cm	102.2 ± 7.4	100.4 ± 7.3	0.290
Fat percent, %	28.1 ± 4.4	27.9 ± 3.9	0.897
BMR, kj/m^2^·h	1503.6 ± 229.2	1483.1 ± 245.8	0.719
**Week 4**			
Height, cm	165.3 ± 7.6	164.2 ± 9.2	0.944
Body weight, kg	72.0 ± 11.6	72.9 ± 13.2	0.020
BMI, kg/m^2^	26.2 ± 2.6	26.8 ± 2.6	0.027
Waist circumference, cm	89.6 ± 9.8	89.5 ± 10.3	0.494
Hip circumference, cm	101.8 ± 7.5	100.5 ± 7.5	0.387
Fat percent, %	27.4 ± 4.5	27.7 ± 4.4	0.634
BMR, kj/m^2^·h	1490.3 ± 231.5	1497.9 ± 264.1	0.021

Week 4 vs. baseline	Difference	*p*-value	Difference	*p*-value
Height, cm	−0.1 (−0.3, 0.2)	0.715	−0.04 (−0.1, 0.01)	0.103
Body weight, kg	−2.2 (−3.4, −1.0)	0.001	−0.1 (−1.0, 0.8)	0.858
BMI, kg/m^2^	−0.8 (−1.2, −0.3)	0.001	−0.04 (−0.4, 0.3)	0.839
Waist circumference, cm	−0.7 (−1.5, 0.1)	0.079	−0.3 (−1.1, 0.4)	0.383
Hip circumference, cm	−0.6 (−1.3, 0.1)	0.086	−0.3 (−0.8, 0.2)	0.300
Fat percent, %	−0.7 (−1.5, 0.03)	0.059	−0.4 (−1.2, 0.4)	0.337
BMR, kj/m^2^·h	−23.9 (−37.7, −10.1)	0.001	−0.3 (−10.0, 9.3)	0.948

BMI, body mass index; BMR, basal metabolic rate; CI, confidence interval. Data are presented as mean ± standard deviation. Differences between study groups at each visit were evaluated using analysis of (co)variance. Analysis of post-intervention data adjusted for baseline measurements. Within-group differences between post-intervention and baseline measurements are presented as the difference of least-squares means (95% CI) and evaluated using paired *t*-test.

### 3.8. Gut microbial diversity

We detected a back-transformed mean increase of ASV relative abundance in Bifidobacterium of 4.5% (*p* = 0.001) and in Lactobacillus of 1.6% (*p* > 0.05) with 4 weeks of probiotics supplementation vs. 0.9% and 0.5% in the control group, respectively (Supplementary Figure 1). There was 2.2% higher abundance (*p* = 0.06) compared to baseline remained for Bifidobacterium at one week after the end of probiotics supplementation, while the abundance of Lactobacillus was 1.6% (*p* > 0.05) higher than the baseline level.

The 20 most abundant ASVs in the FC and FDr probiotics groups are presented in Supplementary Figure 2. After 4 weeks of probiotics intervention, relative abundance decreased from baseline for genus Escherichia-Shigella, increased for Prevotella, Blautia and Klebsiella, and remained similar level for Bacteroides among FC subjects. One week after the end of intervention, the abundance of Prevotella and Blautia among FC subjects with probiotics intake were still higher than their baseline levels; the abundance of Escherichia-Shigella further reduced and that of Klebsiella and Bacteroides were also lower than the baseline level. Among FDr subjects, the probiotics group showed increased mean abundance in Escherichia-Shigella and Blautia, parallel with decreased abundance in Klebsiella, Prevotella and Bacteroides. One week after the end of intervention, these subjects had higher abundance than baseline in all genera analyzed except Blautia. Although some FDr subjects in the probiotics group presented with high abundance of Escherichia-Shigella which brought the mean up, median abundance of Escherichia-Shigella decreased from baseline at both week 4 (−0.36%) and week 5 (−0.42%).

### 3.9. Adverse events

There were a total of 15 (10.7%) AEs during the study, among which 7 (10.0%) in the probiotics group and 8 (11.4%) in the CG. The AEs reported included otitis media (3), traumatism (4), stiff neck (2), cold (2), eczema (2), vaginal infection (1) and conjunctivitis (1). The list of AEs by study group are presented in Supplementary Table 3. None of the AEs was related to the study product, and there was no significant difference in the incidence of AEs between study groups (*p* = 0.595). No serious adverse events occurred during the study.

## 4. Discussion

FC and FDr are two common type of FBDs that can affect men and women of all ages. Various GI symptoms are involved with FC or FDr and may have a significant impact on health and quality of life. The digestive tract contains billions of bacteria from more than 400 different species, both harmful and beneficial, that play an important role in promoting a healthy digestive system by stimulating immune responses and controlling pathogenic mechanisms (42). Recently, there has been a growing interest in using probiotics to enhance GI health.

In the present study, we conducted two double-blinded, randomized, and parallel-controlled trials to evaluate the efficacy of a multi-strain probiotic formula on FC and FDr in Chinese adults. Each of the strains in this formula has been studied alone or in combination with some other strains and have well established health benefits. Among them, *B. lactis* Bi-04, *L. acidophilus* NCFM, *B. lactis* HN019 and *L. plantarum* Lp-115 have been shown to enhance immunity in different age groups (43–48). *Bifidobacterium lactis* Bl-04 demonstrates effect in reducing symptoms of diarrhea (49). *Lacticaseibacillus paracasei* Lpc-37 has proven effect in preventing chronic stress-associated behaviors (50). The primary health attributes of *B. lactis* B420 are weight management and metabolic health, which may be related to its ability to support intestinal barrier integrity (51, 52). The combination of *L. acidophilus* NCFM and *B. lactis* HN019 could help to shorten colon transit time (53).

As two types of bow disorders, FC and FDr are linked to some common GI symptoms. Our results showed that 4 weeks of supplementation with the probiotic formula under study effectively relieved bloating, abdominal pain, early feeling of fullness, dyspepsia and improved satisfaction with digestive function in most FC and FDr subjects. It also markedly relieved belching, poor appetite, heavy stomach and poor GI motility in subjects with FC. The probiotic effect on these symptoms presented since the second week of the intervention and extended through the washout period. Self-assessed intestinal health improved since week 2 in the FC group and at week 4 in the FDr group. These results indicate that the probiotics formula is effective for improving overall gut health and may benefit individuals with a wide range of GI issues. In both sub-trials, the study probiotic formula showed accelerating beneficial effect on the satisfaction with defecation habits from week 2 to week 4 of supplementation.

Overall, the study probiotics formula regulated the weekly frequency, consistency and color of stool toward the normal levels in both FC and FDr subjects. The stool form of the FC probiotics gradually changed from on average lumpy sausage-shaped toward smoother and softer sausage-shaped, and their stool color became lighter indicating increased moisture in the stool. Subjects with FDr had more formed and darker-colored stools after probiotics consumption. Both scores of stool consistency and stool color fell near the middle range of the Bristol scale. These beneficial effects remained within 1 week after discontinuation of probiotics consumption. Our findings support the claims from previous studies about the beneficial effects of probiotics in treating intestinal disorders and maintaining the health of the intestinal tract (53–56). For subjects with FC, mean stool frequency increased significantly from less than four times to more than six times per week at the end of probiotics intervention. This result is in line with that from a previous randomized control trail, which showed *B. lactic* HN019 to promote bowel movement frequency (+2 times/week) in FC participants with low stool frequency (57). In addition to *B. lactic* HN019, *L. acidophilus* NCFM also yielded encouraging results in the alleviation of slow stool transit. A recent study investigated this potential on subjects with constipation using a yogurt mixed with these two strains and a soluble fiber. After 14 days of supplementation, the probiotics group showed a significantly shortened colon transit time (53). Meanwhile, less FDr subjects in our study had multiple defecations in 1 day following probiotics consumption. Two other studies have shown similar findings where both *L. acidophilus* NCFM and *B. lactis* Bi-07 contributed to reduce the incidence and frequency of episodes of diarrhea (58, 59), suggesting probiotics’ benefit of providing regulation of GI disorders.

The relationship between intestinal health and a person’s emotional state is bi-directional. Any chronic illness has psychosocial consequences on one’s general well-being, sense of control over the symptoms, and implications of the illness in terms of daily function status (1). These psychosocial effects of illness may in tern exacerbating symptoms and leading to anxiety. On the other hand, psychological stress or one’s emotional state sends signals to and from the nervous system through the vagus nerve, further damaging the integrity of the microbiome, and subsequently lead to irregular motility throughout the GI tract and generate gastrointestinal symptoms (60). By alternating gut microbiome, probiotics execute their functions through the brain-gut axis. Regulating the gut microbial composition may not only improve intestinal health, but can have systemic effects including mental wellbeing, metabolic health and much more. As shown in our study, the improvement of intestinal health was accompanied by reduced anxiety, depression and perceived stress in both FC and FDr subjects.

Possible mechanisms of action through which probiotics can impact human health include inhibition of pathogenic bacteria, immunomodulatory effects, stimulation of barrier function and metabolic function (60). Inflammation in patients with diarrhea is high (25, 26). As shown by our study, the significant reduction in the level of IL-4 in the FDr probiotics group indicated that probiotics intervention had a better regulation on this inflammation marker. Patients with chronic constipation likely have dysbiosis in large bowel, including a relative decrease of beneficial bacteria and a parallel increase of potentially pathogenic microorganisms. This condition also causes low-grade inflammation (27). The hsCRP test measures even low levels of inflammation. We detected significant reductions in hsCRP in the probiotics group of both FC and FDr subjects, adding to existing evidence that probiotic bacteria can stimulate the anti-inflammatory component of the immune system to release cytokines and hormones that disrupt the damaging inflammatory cycle (61, 62). Following probiotics supplementation to both FC and FDr subjects, our study also found a significant increase of IFN-γ and IgA, which act as master regulators of immune responses and inflammation (63, 64), suggesting that the mechanism of action of this probiotic formula may have an immune-modulating effect. Furthermore, fecal sIgA plays an important role in the homeostatic regulation of microbiota. Any potential dysfunctions can lead to the development of pathologies such as inflammatory bowel diseases (65). Supplementation with the study probiotics formula markedly increased fecal sIgA level in both FC and FDr subjects.

Besides directly regulating gut microbiota composition, the effect of probiotics can be mediated by their metabolites, such as SCFAs that may exercise anti-inflammatory effects (66, 67). An earlier research reported that *L. casei* increased the contents of SCFAs when alleviating antibiotic-related diarrhea (49). Supplementation of the probiotic formula in our study for 4 weeks effectively promoted the production of three major SCFAs among FDr subjects. The increase of SCFAs can contribute to the host immune response and inhibiting the production pro-inflammatory cytokines (68, 69). SCFAs mainly come from the fermentation of indigestible carbohydrate and protein degradation in large intestine and have been shown to have a very positive effect on the energy metabolism (70). A lower abundance of specific bacteria and SCFAs lead to gut barrier dysfunction, low-grade inflammation and further to altered lipids (71), and energy homeostasis are characteristic for obesity (72). Our study also showed that probiotics intake promoted the utilization of acetate, propionate, and butyrate acid in the FC group, thereby improving gut barrier function. The changes in SCFAs following probiotics intervention are of similar levels for both FC and FDr subjects. This is probably related to the small addition of prebiotic in our study formula. According to the researchers in Japan, relatively low amounts of prebiotics can increase the body’s production of SCFAs by activating the metabolism of human colonic microbiota (73). Adding prebiotics to the supplementation is a promising microbiota-targeting approach for promoting health (74).

For these subjects with FC and elevated BMI (>24 kg/m^2^), the probiotics formula showed beneficial effect in weight control and lipid metabolism. Significantly increased SCFAs and reduced waist circumference in overweight and obese adults following the supplementation of probiotics together with a dietary fiber have been documented by another study (75). While a previous randomized crossover trial with supplementation of *B. lactis* 420 combined with *L. acidophilus* 74-2 showed that serum cholesterol levels were not influenced by the probiotics and serum TG concentration decreased significantly by 11.6% in the probiotic supplementation period (76), we observed significant improvement in serum TC, TG and HDL-C levels. As four among the six probiotic strains in our study formula have been shown in previous studies to support metabolic health (52, 77–79), the combination of these strains may have additive or synergistic effects and require further exploration.

All probiotic strains used in this formula have researches demonstrating their safety, stability and survival to reach the gut alive. These strains have been recovered in feces in significantly elevated number after supplementation, suggesting successful passage through digestive system (76, 80–86). Monitoring the changes in the gut microbiome after probiotic intake can provide a better understanding of the mechanisms underlying its health benefits. It is worth noting that following 4 weeks of probiotics consumption in our study, relative abundance of Bifidobacterium was greatly improved. The abundance Lactobacillus also increased, although maybe due to the small sample size of our microbial analysis, the difference compared to baseline was not significant. These findings indicated good survival of the probiotic strains in this study formula in the upper GI tract conditions during their passage toward the colon.

To the best of our knowledge, this study is the first to investigate one multi-strain probiotic formula in both the FC and the FDr subjects identified by Rome IV criteria. As FC and FDr shared various gastrointestinal symptoms, we designed the study with two randomized trials to investigate the probiotics effect in both population parallelly. In addition to assessing the effect of this formula on self-reported symptoms and defecation habits, we used psychological and physiological makers to evaluate other potential effects which may be related to FC or FDr. Despite all the positive outcomes found in the present study, some limitations must be acknowledged. The gut microbial exploratory analysis was performed for a small subsample. Additional researches are required with larger sample size due to the immense diversity among the microbiome of individuals. Moreover, although the effect of probiotics extended 1 week after discontinuation of supplementation, we do not have data to support the hypothesis that the modification of gut microbiota might persist beyond the period of observation. We would like to address these limitations in future studies.

## 5. Conclusion

In conclusion, our findings suggest that twice-daily consumption of the WONDERLAB® probiotics formula regulates the balance of the gut microbiota and may be beneficial in relieving FC and FDr related GI symptoms, improving defecation habits and satisfaction, normalizing stool frequency and stool characteristics, reducing negative emotional feeling and perceived stress, modulating immune response, and promoting bacteria metabolism. In addition, probiotics supplementation might assist in weight control and improve lipid profile in slightly overweight subjects with FC.

## Data availability statement

The raw data supporting the conclusions of this article will be made available by the authors, without undue reservation.

## Ethics statement

The studies involving human participants were reviewed and approved by Institutional Review Board of Shanghai Nutrition Society. The patients/participants provided their written informed consent to participate in this study.

## Author contributions

YZ, LX, and SZ designed and conducted the research. YL conducted data curation. JN performed the formal analysis. YZ wrote the manuscript. LX and GX supervised the research and reviewed and edited the manuscript. All authors contributed to the article and approved the submitted version.

## Funding

The study was funded by Shenzhen Precision Health Food Technology Co., Ltd.

## Conflict of interest

YZ, SZ, and GX were full-time employees of Shenzhen Precision Health Food Technology Co., Ltd., which is the funding source of this study. JN was an employee of Sprim (China) Consulting Co., Ltd., which is the company hired by Shenzhen Precision Health to conduct the study.

The authors declare that this study received funding from Shenzhen Precision Health Food Technology Co., Ltd. The funder had the following involvement in the study: study design, interpretation of data, the writing of this article and the decision to submit it for publication.

## Publisher’s note

All claims expressed in this article are solely those of the authors and do not necessarily represent those of their affiliated organizations, or those of the publisher, the editors and the reviewers. Any product that may be evaluated in this article, or claim that may be made by its manufacturer, is not guaranteed or endorsed by the publisher.

## Abbreviations

AE: Adverse event
ASV: Amplicon sequence variants
BMI: Body mass index
CG: Control group
CI: Confidence interval
FBDs: Functional bowel disorders
FC: Functional constipation
FDr: Functional diarrhea
GI: Gastrointestinal
HDL-C: High-density lipoprotein cholesterol
hsCRP: High-sensitivity C-reactive protein
IFN-γ: Interferon-γ
IgE: Immunoglobulin E
IgG: Immunoglobulin G
IgM: Immunoglobulin M
IL: Interleukin
LDL-C: Low-density lipoprotein cholesterol
MET: Metabolic equivalent of task
PSS: Perceived stress scale
SAE: Serious adverse event
SCFAs: Short-chain fatty acids
TC: Total cholesterol
TG: Triglyceride
