# Supplementary material for: Effect of a probiotic formula on gastrointestinal health, immune responses and metabolic health in adults with functional constipation or functional diarrhea
Source: Front Nutr. 2023 Jul 10;10:1196625. doi: 10.3389/fnut.2023.1196625 (PMC10368241; doi:10.3389/fnut.2023.1196625)
Supplement: Supplementary file 1 [file Data_Sheet_1.docx]

Supplementary Material

Effect of a probiotic formula on gastrointestinal health, immune responses and metabolic health in adults with functional constipation or functional diarrhea

Yanyi Zheng, Leiming Xu*, Silu Zhang, Yanwen Liu, Jiayi Ni, and Guoxun Xiao*

*** Correspondence:** Leiming Xu: leiming.xu@aliyun.com; Guoxun Xiao: shawn@wonderlab.top

# Supplementary Figures and Tables

## Supplementary Figures


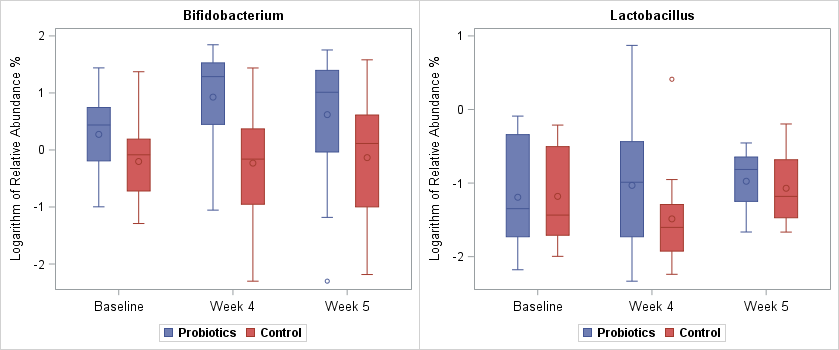


**Supplementary Figure 1.** Box plot of amplicon sequence variants (ASV) relative abundance % (log10 scale) of Bifidobacterium and Lactobacillus in a pooled subsample of subjects with functional constipation or functional diarrhea by fecal 16sRNA sequencing.

**
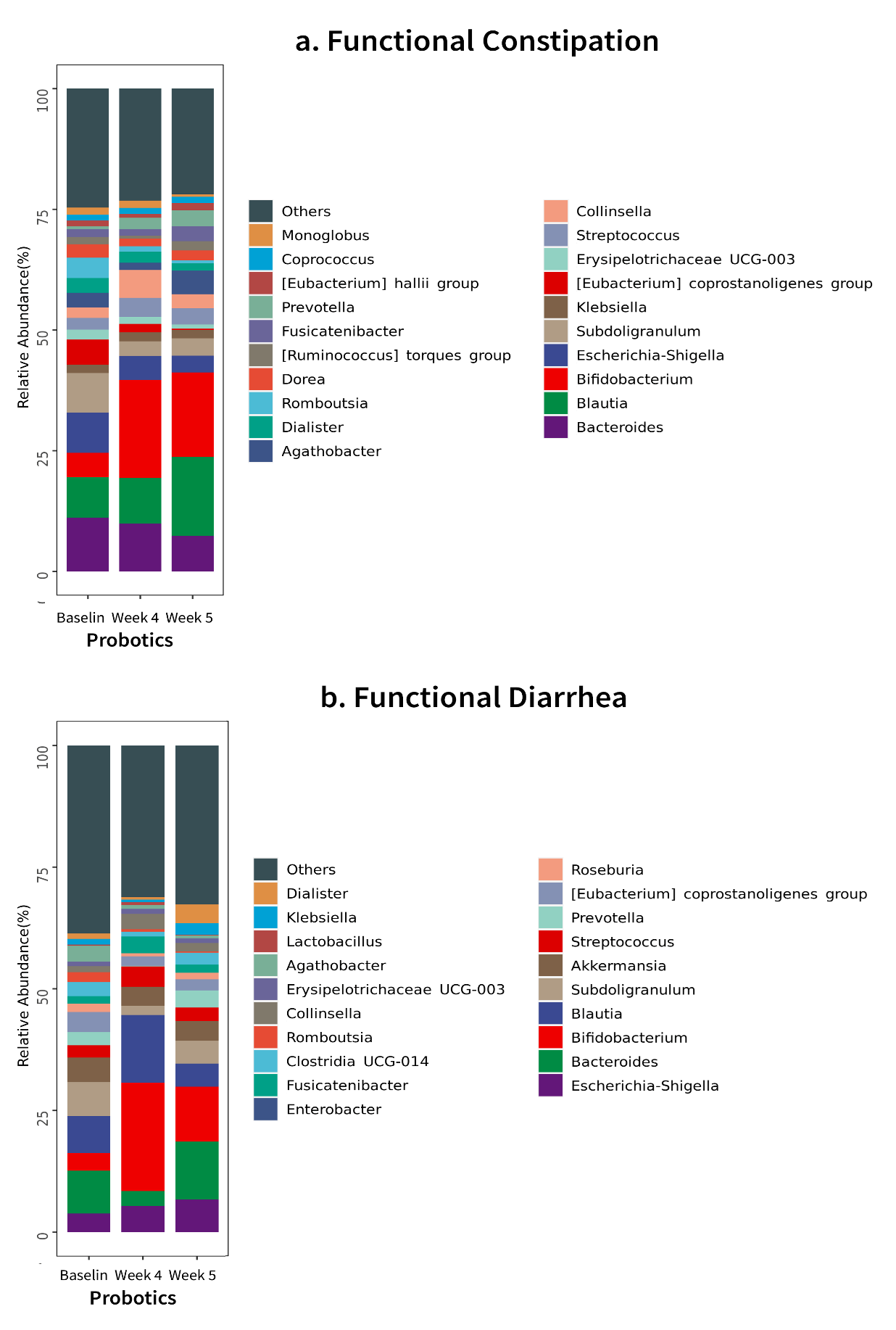
**

**Supplementary Figure 2.** Relative abundance of twenty most abundant amplicon sequence variants (ASV) in the probiotics group by amplicon analysis of 16S rRNA genes. The ASVs were identified at the lowest possible classification and summarized by genus.

## Supplementary Tables

**Supplementary Table 1.** Daily Food Intake by 72-hour Dietary Recall

| **Visit** | **Outcome** | **Functional Constipation** | | | **Functional Diarrhea** | | |
| --- | --- | --- | --- | --- | --- | --- | --- |
|  |  | **Probiotics** | **Control** | **Group Difference p-value** | **Probiotics** | **Control** | **Group Difference p-value** |
| Baseline | Diary product, ml/day | None | None | 1.000 | None | None | 1.000 |
|  | Cheese, time/day | None | None | 1.000 | None | None | 1.000 |
|  | Fruit, g/day | 433.3 (300, 600) | 406.7 (300, 733.3) | 0.933 | 400 (266.7, 566.7) | 433.3 (300, 700) | 0.557 |
|  | Fruit juice, cup/day | 0 (0, 0) | 0 (0, 0) | 0.816 | 0 (0, 0) | 0 (0, 0) | 0.236 |
|  | Vegetable, g/day | 200 (133.3, 233.3) | 166.7 (133.3, 233.3) | 0.462 | 133.3 (133.3, 200) | 166.7 (133.3, 233.3) | 0.813 |
|  | Egg and meat, g/day | 200 (166.7, 200) | 200 (166.7, 233.3) | 0.743 | 200 (166.7, 233.3) | 200 (166.7, 233.3) | 0.871 |
|  | Cereal, g/day | 300 (300, 300) | 300 (300, 300) | 1.000 | 300 (300, 300) | 300 (300, 300) | 1.000 |
|  | Beverage, ml/day | 80 (0, 240) | 80 (0, 240) | 0.823 | 0 (0, 80) | 0 (0, 160) | 0.375 |
|  | Seasoning, time/day | 6 (6, 6) | 6 (6, 6) | 0.815 | 6 (6, 6) | 6 (6, 6) | 0.668 |
| Week 4 | Diary product, ml/day | None | None | 1.000 | None | None | 1.000 |
|  | Cheese, time/day | None | None | 1.000 | None | None | 1.000 |
|  | Fruit, g/day | 466.7 (333.3, 700) | 416.7 (300, 633.3) | 0.767 | 435 (320, 633.3) | 433.3 (300, 733.3) | 0.847 |
|  | Fruit juice, cup/day | 0 (0, 0) | 0 (0, 0) | 0.736 | 0 (0, 0) | 0 (0, 0) | 0.164 |
|  | Vegetable, g/day | 166.7 (133.3, 233.3) | 166.7 (133.3, 200) | 0.931 | 133.3 (133.3, 200) | 166.7 (133.3, 200) | 0.745 |
|  | Egg and meat, g/day | 200 (200, 233.3) | 200 (200, 266.7) | 0.492 | 183.3 (166.7, 200) | 200 (166.7, 200) | 0.334 |
|  | Cereal, g/day | 300 (300, 300) | 300 (300, 300) | 1.000 | 300 (300, 300) | 300 (300, 300) | 1.000 |
|  | Beverage, ml/day | 80 (0, 240) | 80 (0, 240) | 0.560 | 0 (0, 0) | 0 (0, 80) | 0.481 |
|  | Seasoning, time/day | 6 (6, 6) | 6 (6, 6) | 0.665 | 6 (6, 6) | 6 (6, 6) | 0.096 |
| Week 5 | Diary product, ml/day | None | None | 1.000 | None | None | 1.000 |
|  | Cheese, time/day | None | None | 1.000 | None | None | 1.000 |
|  | Fruit, g/day | 433.3 (333.3, 566.7) | 466.7 (300, 633.3) | 0.793 | 400 (236.7, 516.7) | 466.7 (366.7, 633.3) | 0.156 |
|  | Fruit juice, cup/day | 0 (0, 0) | 0 (0, 0) | 0.769 | 0 (0, 0) | 0 (0, 0) | 0.187 |
|  | Vegetable, g/day | 200 (133.3, 233.3) | 183.3 (133.3, 233.3) | 0.570 | 166.7 (133.3, 233.3) | 166.7 (133.3, 233.3) | 0.686 |
|  | Egg and meat, g/day | 200 (166.7, 200) | 200 (133.3, 233.3) | 0.875 | 183.3 (133.3, 200) | 200 (133.3, 200) | 0.754 |
|  | Cereal, g/day | 300 (300, 300) | 300 (300, 300) | 1.000 | 300 (300, 300) | 300 (300, 300) | 1.000 |
|  | Beverage, ml/day | 80 (0, 240) | 200 (80, 240) | 0.734 | 0 (0, 80) | 0 (0, 80) | 0.936 |
|  | Seasoning, time/day | 6 (6, 6) | 6 (6, 6) | 0.875 | 6 (6, 6) | 6 (6, 6) | 0.074 |
|  |  | **Within-Group Difference p-value** | **Within-Group Difference p-value** |  | **Within-Group Difference p-value** | **Within-Group Difference p-value** |  |
| Week 4 vs. Baseline | Diary product, ml/day | None | None |  | None | None |  |
|  | Cheese, time/day | None | None |  | None | None |  |
|  | Fruit, g/day | 0.843 | 0.647 |  | 0.658 | 0.748 |  |
|  | Fruit juice, cup/day | 1.000 | 0.750 |  | 1.000 | 0.500 |  |
|  | Vegetable, g/day | 0.908 | 0.680 |  | 0.721 | 0.382 |  |
|  | Egg and meat, g/day | 0.347 | 0.154 |  | 0.359 | 0.823 |  |
|  | Cereal, g/day | 1.000 | 1.000 |  | 1.000 | 1.000 |  |
|  | Beverage, ml/day | 0.661 | 0.141 |  | 0.748 | 0.384 |  |
|  | Seasoning, time/day | 1.000 | 0.625 |  | 0.688 | 0.250 |  |
| Week 5  vs.  Baseline | Diary product, ml/day | None | None |  | None | None |  |
|  | Cheese, time/day | None | None |  | None | None |  |
|  | Fruit, g/day | 0.920 | 0.825 |  | 0.960 | 0.651 |  |
|  | Fruit juice, cup/day | 0.984 | 1.000 |  | 0.805 | 1.000 |  |
|  | Vegetable, g/day | 0.187 | 0.649 |  | 0.334 | 0.436 |  |
|  | Egg and meat, g/day | 0.361 | 0.202 |  | 0.615 | 0.407 |  |
|  | Cereal, g/day | 1.000 | 1.000 |  | 1.000 | 1.000 |  |
|  | Beverage, ml/day | 0.228 | 0.223 |  | 0.622 | 0.468 |  |
|  | Seasoning, time/day | 0.500 | 0.375 |  | 0.250 | 1.000 |  |

Data are presented as median (1st and 3rd quantile); inter-group differences are tested by Kruskal Wallis test; intra-group difference are tested by Wilcoxon signed-rank test.

**Supplementary Table 2.** Weekly Physical Exercise

| **Visit** | **Outcome** | **Functional Constipation** | | | **Functional Diarrhea** | | |
| --- | --- | --- | --- | --- | --- | --- | --- |
|  |  | **Probiotics** | **Control** | **Group Difference p-value** | **Probiotics** | **Control** | **Group Difference p-value** |
| Baseline | Vigorous MET-minutes/week ^a^ | 0 (0, 960) | 0 (0, 960) | 0.781 | 0 (0, 1280) | 0 (0, 1080) | 0.918 |
|  | Moderate MET-minutes/week ^a^ | 480 (0, 1440) | 480 (0, 1200) | 0.752 | 480 (60, 1200) | 480 (0, 840) | 0.803 |
|  | Walk MET-minutes/week (MET) ^a^ | 693 (594, 1732.5) | 1188 (693, 1650) | 0.406 | 792 (495, 1386) | 693 (462, 1386) | 0.854 |
|  | Total MET-minutes/week ^a^ | 2346 (840, 3093) | 1986 (1116, 3426) | 0.485 | 2025 (1173, 3177) | 2293 (1022, 3570) | 0.883 |
|  | Average daily duraiton of sitting, hour | 5.63±2.46 | 5.53±2.35 | 0.859 | 5.77±2.55 | 5.67±2.25 | 0.856 |
| Week 4 | Vigorous MET-minutes/week ^a^ | 0 (0, 720) | 0 (0, 1440) | 0.916 | 0 (0, 960) | 0 (0, 960) | 0.779 |
|  | Moderate MET-minutes/week ^a^ | 320 (0, 840) | 480 (0, 960) | 0.709 | 360 (0, 1200) | 240 (0, 840) | 0.718 |
|  | Walk MET-minutes/week (MET) ^a^ | 792 (462, 1386) | 1386 (396, 1584) | 0.501 | 693 (346.5, 1386) | 808.5 (462, 1485) | 0.517 |
|  | Total MET-minutes/week ^a^ | 1836 (792, 3612) | 1746 (396, 3885) | 0.934 | 1775 (693, 2880) | 2015 (1116, 2772) | 0.846 |
|  | Average daily duraiton of sitting, hour | 5.40±2.76 | 5.05±2.67 | 0.612 | 5.84±2.90 | 5.68±2.84 | 0.819 |
| Week 5 | Vigorous MET-minutes/week ^a^ | 0 (0, 640) | 0 (0, 0) | 0.691 | 0 (0, 1440) | 0 (0, 480) | 0.965 |
|  | Moderate MET-minutes/week ^a^ | 480 (0, 1200) | 240 (0, 1600) | 0.711 | 720 (0, 1080) | 480 (0, 1200) | 0.705 |
|  | Walk MET-minutes/week (MET) ^a^ | 990 (660, 1386) | 792 (495, 1386) | 0.500 | 1155 (396, 1386) | 1155 (462, 1386) | 0.859 |
|  | Total MET-minutes/week ^a^ | 2373 (1074, 3279) | 1752 (693, 4194) | 0.837 | 2079 (1038, 2970) | 1485 (1008, 3135) | 0.837 |
|  | Average daily duraiton of sitting, hour | 5.45±2.42 | 5.23±3.27 | 0.760 | 5.77±2.75 | 5.65±1.87 | 0.840 |
|  |  | **Within-Group Difference p-value** | **Within-Group Difference p-value** |  | **Within-Group Difference p-value** | **Within-Group Difference p-value** |  |
| Week 4 vs. Baseline | Vigorous MET-minutes/week ^a^ | 0.406 | 0.781 |  | 0.443 | 0.429 |  |
|  | Moderate MET-minutes/week ^a^ | 0.300 | 0.413 |  | 0.612 | 0.656 |  |
|  | Walk MET-minutes/week (MET) ^a^ | 0.818 | 0.820 |  | 0.526 | 0.602 |  |
|  | Total MET-minutes/week ^a^ | 0.371 | 0.712 |  | 0.263 | 0.896 |  |
|  | Average daily duraiton of sitting, hour | 0.828 | 0.238 |  | 0.887 | 0.645 |  |
| Week 5 vs. Baseline | Vigorous MET-minutes/week ^a^ | 0.797 | 0.511 |  | 0.530 | 0.684 |  |
|  | Moderate MET-minutes/week ^a^ | 0.573 | 0.566 |  | 0.916 | 0.788 |  |
|  | Walk MET-minutes/week (MET) ^a^ | 0.886 | 0.546 |  | 0.935 | 0.621 |  |
|  | Total MET-minutes/week ^a^ | 0.854 | 0.854 |  | 0.535 | 0.985 |  |
|  | Average daily duraiton of sitting, hour | 0.915 | 0.531 |  | 1.000 | 0.569 |  |
| Week 5 vs. Week 4 | Vigorous MET-minutes/week ^a^ | 0.813 | 0.609 |  | 0.923 | 0.508 |  |
|  | Moderate MET-minutes/week ^a^ | 0.468 | 0.593 |  | 0.898 | 0.101 |  |
|  | Walk MET-minutes/week (MET) ^a^ | 0.977 | 0.857 |  | 0.490 | 0.916 |  |
|  | Total MET-minutes/week ^a^ | 0.694 | 0.925 |  | 0.909 | 0.818 |  |
|  | Average daily duraiton of sitting, hour | 0.928 | 0.779 |  | 0.916 | 0.959 |  |

MET: metabolic equivalent of task.

Unless otherwise noted, data are summarized by mean±standard deviation. Differences between study groups were evaluated using one-way analysis of variance. Within-group differnces were evaluated using paired t-test.

^a^Dare are presented as median (1st and 3rd quantile). Differences between groups were evaluated using Kruskal Wallis test. Within-group differences were evaluated using Wilcoxon signed-rank test.

**Supplementary Table 3.** List of Adverse Events

| **Adverse Event** | **Functional Constipation and Functional Diarrhea** | | |  |
| --- | --- | --- | --- | --- |
|  | **Probiotics Group (n=70)** | **Control Group (n=70)** | **Overall (n=140)** | |
| EENT 001 Otitis media | 1 (1.4) | 2 (2.9) | 3 (2.1) | |
| MS 003 Traumatism | 3 (4.3) | 1 (1.4) | 4 (2.9) | |
| MS 005 Stiff neck | 2 (2.9) | 0 (0.0) | 2 (1.4) | |
| RESP 001 Cold | 0 (0.0) | 2 (2.9) | 2 (1.4) | |
| SK 004 Eczema | 0 (0.0) | 2 (2.9) | 2 (1.4) | |
| UG 004 Vaginal infection | 1 (1.4) | 0 (0.0) | 1 (0.7) | |
| EENT 006 Conjunctivitis | 0 (0.0) | 1 (1.4) | 1 (0.7) | |
| **Total** | **7 (10.00)** | **8 (11.4)** | **15 (10.7%)**  **Group difference p-value: 0.595** | |

Descriptive statistics are presented as frequency (%). Fisher's exact test was used to compare the differences between groups.
